# Genome-wide analysis of haplotype interaction for the data from the North American Rheumatoid Arthritis Consortium

**DOI:** 10.1186/1753-6561-3-s7-s34

**Published:** 2009-12-15

**Authors:** Jungsun Park, Junghyun Namkung, Mina Jhun, Taesung Park

**Affiliations:** 1 Interdisciplinary Program in Bioinformatics, Seoul National University, San56-l, Shin Lim-Dong, Kwan Ak-Ku, Seoul 151-747, Republic of Korea; 2 Department of Statistics, College of Natural Science, Seoul National University, San56l, Shin Lim-Dong, Kwan Ak-Ku, Seoul 151-747, Republic of Korea

## Abstract

Recent genome-wide association studies on several complex diseases have focused on individual single-nucleotide polymorphism (SNP) analysis; however, not many studies have reported interactions among genes perhaps because the gene-gene and gene-environment interaction analysis could be infeasible due to heavy computing requirements. In this study we propose a new strategy for exploring the interactions among haplotypes. The proposed method consists of two steps. Step 1 tests the single-SNP association of whole genome with multiple testing corrections and finds the haplotype blocks of the significant SNPs. Step 2 performs interaction analysis of haplotypes within blocks. Our proposed method is applied to the rheumatoid arthritis data for Genetic Analysis Workshop 16.

## Background

Complex diseases such as rheumatoid arthritis (RA) are the results of a complex interplay of genetic and environmental factors. Previously, a linkage study of Jawaheer et al. [1] showed that regions from chromosome 1 interact with chromosome 6, resulting in synergistic effect on risk of RA. Simulation studies showed that interaction analysis can be more powerful to detect disease associated genes than analysis ignoring interactions even after correction for multiple testing [2,3]. Methods based on haplotypes were also shown to provide additional power for mapping disease genes when compared with the analysis of individual single-nucleotide polymorphisms (SNPs) [4,5]. Because a haplotype comprises multiple SNPs on the same inherited chromosomes, the haplotype-based approaches can also provide insight into factors influencing the dependency among genetic markers. Such insight may provide information essential for understanding human evolution and also for identifying *cis*-interactions between two or more causal variants [6].

Recently, genome-wide association studies (GWAS) on several complex diseases were successfully conducted and some promising results with putative disease-related genes were reported [7-9]. However, analyses from those studies only considered individual SNPs, and not many studies have reported interactions among genes in GWAS because the gene-gene and gene-environment interaction analysis could be infeasible due to heavy computing times. For example, the North American Rheumatoid Arthritis Consortium (NARAC) data for the genetic data analysis workshop (GAW16) contains 545,080 SNP-genotype fields from the Illumina 550 k chip. Note that more than 10^11 ^interaction models are possible for all pairs of SNPs in this data set, which requires enormous computing times.

In this study, we propose genome-wide analysis of haplotype interaction (GWAHI) to discover interactions between unlinked regions based on haplotypes. The proposed GWAHI consists of the following two steps: Step 1 tests the single-SNP association of whole genome with multiple testing corrections and finds the haplotype blocks of the significant SNPs, and Step 2 performs interaction analysis of haplotypes within blocks. Our proposed GWAHI is applied to the RA data for GAW16.

## Methods

### Data

In the GAW16 RA data, genotypes for 545,080 SNPs from total 2,062 individuals were provided. The samples consisted of 868 patients and 1,194 controls. We excluded outliers of population stratification test performed by Egenstrat [10] and samples showing sex matching error tested by PLINK [11]. We also excluded SNPs with minor allele frequency less than 5% or with the significance level of 10^-6 ^for Hardy-Weinberg equilibrium test. After data cleaning, 491,345 SNPs and 2,048 samples were used in our analysis.

### Single-SNP association test

We tested for additive and co-dominant genetic models using PLINK [11]. For the additive model we used logistic--genotypic option in PLINK, which provided a test based on logistic regression; for the co-dominant model we used model option in PLINK, which provided a χ^2 ^test. Both tests were performed over the whole genome. For multiple-testing correction, we applied the Bonferoni correction. SNPs were selected if they showed significant association results with *p*-values less than 1.03 × 10^7^.

### Haplotype block construction and association test

We constructed haplotype blocks for each chromosome using Haploview [12] and Gabriel's block definition. Next, we identified haplotype blocks containing one or more SNPs chosen from the individual SNP test. We also included haplotype blocks that showed significant association (*p*-value < 5.35 × 10^-7^). The haplotype association test was also performed by PLINK [11]. We used hap-assoc option in PLINK, which generated haplotype-specific tests (1 df).

### Haplotype interaction

Association tests using haplotype interaction were conducted using the method introduced by Becker et al. [3] and implemented in program FAMHAP. FAMHAP uses the expectation-maximization algorithm to obtain maximum-likelihood estimates of the haplotype frequencies at each of the unlinked regions for pooled case-control samples. Then, a contingency table was constructed that comprises rows referring to disease status and columns referring to multi-region haplotype configurations. For association tests, the usual chi-square tests and permutation tests were performed [3].

## Results

### Single-SNP and haplotype association analysis

**Figure 1:**
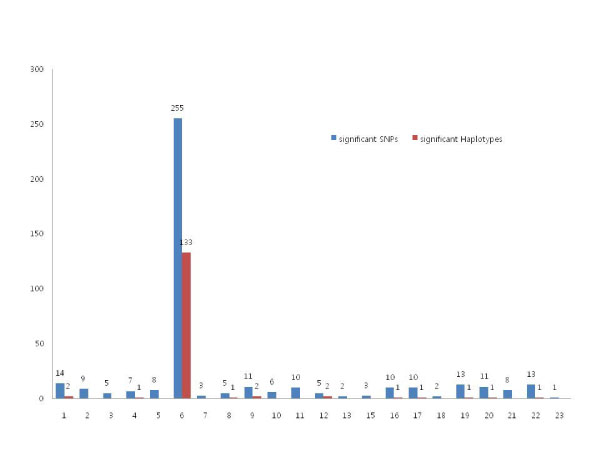
**The proportion of significant SNPs and haplotype blocks from the single association tests**. In our single association analysis, 62% of the significant SNP and 91% of the significant haplotypes were located in chromosome 6. There were no significant markers in chromosome 14.

SNP and haplotype association analysis identified 411 significant SNPs and 146 haplotype blocks, among which 255 SNPs and 133 haplotypes were from chromosome 6. The distribution of the significant SNPs over the chromosome is shown in Figure 1. Among 411 SNPs, 219 SNPs overlapped and were included in the identified haplotype blocks. In order to see how much influence the significant SNPs have on the significance of the haplotype blocks, we calculated the correlation coefficient between the *p*-values of overlapped SNPs and haplotype blocks. After the log-transformation of *p*-values, the correlation coefficients were 0.7295 (*p*-value < 1.0 × 10^-15^) for the additive model and 0.8036 (*p*-value < 1.0 × 10^-15^) for the codominant model. Thus, we conclude that the significant SNPs have much influence on the significance of the haplotype blocks. We also computed the correlation coefficients focusing only on chromosome 6. For the 211 overlapped SNPs, we obtained very similar correlation coefficients. When we mapped the significant SNPs using dbSNP database [13], 238 unique genes or putative genes were identified. Many SNPs and blocks were located in the human leukocyte antigen (HLA) regions, as presented in Table 1. Some of our results reconfirmed the previous findings on genetic factors attributable to RA risk reported in the OMIM database [14]. For example, *PTPN22 *on chromosome 1, *TRAF1 *on chromosome 9, and *NFKBIL1*, *HLA-C*, and *HLA-G *on chromosome 6 were also detected by our association analysis. Pathway mapping of the 238 identified genes showed that 51 genes were matched with 60 KEGG pathways (Additional File 1), among which 18 KEGG pathways were related to immune responses and signaling pathways.

**Table 1:** The genes containing significant SNPs and haplotype blocks and comprising the HLA regions

HLA class	Locus name	No. SNPs	No. haplotypes
Class I	*HLA-C*	1	1
	*HLA-E*	0	1
	*HLA-F*	0	1
	*HLA-G*	1	1
	*HLA-H*	2	2
	*HLA-80*	1	1
			
Class II	*HLA-DMA*	0	1
	*HLA-DMB*	0	1
	*HLA-DOA*	1	2
	*HLA-DOB*	1	2
	*HLA-DPA1*	4	1
	*HLA-DQA1*	0	1
	*HLA-DQA2*	1	1
	*HLA-DQB1*	1	0
	*HLA-DQB2*	0	1
	*HLA-DRA*	0	1
	*HLA-DRB9*	2	1

We identified the functional class of the significant SNPs, among which 23 SNPs were non-synonymous (Table 2). We then tested whether the amino-acid substitution caused by the non-synonymous SNPs affects protein function damaging using the SIFT program. SIFT predicts whether an amino acid substitution affects protein function based on sequence homology and the physical properties of amino acids [15].

**Table 2:** The functional class of significant SNPs

Function class	No. SNPs
Intron	127
UTR	23
Cds-nonsynonymous	23
Cds-synonymous	7
Locus	32

We determined out that two non-synonymous SNPs were not functionally tolerant and were reported as linked to disease in OMIM [14]. One of the non-synonymous SNPs was rs2075800, which was reported to be related with sarcoidosis [16], an immune system disorder characterized by non-caseating granulomas [17]. The other SNP was rs2476601 in *PTPN22*, which was reported to be associated with four separate autoimmune phenotypes: type I diabetes mellitus, RA, systemic lupus erythematosus, and Hashimoto thyroiditis [18].

### Haplotype block construction and association test

We started to construct haplotype blocks from the shortest chromosome 22 sequentially. Due to lack of memory space in our system (6 GB memory), computation was completed only for the chromosomes up to 18. In order to handle memory problem, we split the whole chromosome into several short regions containing 10,000 SNPs (60 Mb). Each split region was defined to have 500 overlap SNPs (3 Mb) overlapping with its neighbor regions. Then we reconstructed the haplotype blocks for each region and merged them using the overlapping regions. Through the split-and-merge haplotype reconstruction, a total of 93,397 haplotype blocks were constructed. The average block length was 17055.5 base-pairs (median, 7638 base-pairs), and one block included 4.3 SNPs on average (median, 3 SNPs). Finally, 338 haplotype blocks were selected, which contained either significant SNPs or haplotype blocks.

### Haplotype interaction

For Step 2, we conducted the interaction analysis for all pairs of the 338 haplotype blocks. The interaction analysis selected 46,995 haplotype pairs from 56,953 possible pairs (Bonferoni corrected *p*-value < 8.78 × 10^-7^). Among the detected haplotype pairs, 13,563 were from chromosome 6; 25,300 pairs included one haplotype from chromosome 6; 8,132 pairs were not from chromosome 6. We then constructed a haplotype-based network graph in which each node represents a haplotype. This network graph illustrates an overview of haplotype interactions. Each edge represents an interaction of a pair of haplotypes. The more haplotypes connected, the more interactions exist between the haplotypes. We identified 39 hub haplotypes that highly interacted with other haplotypes. Twenty-two of the hub haplotype were located in 15 gene regions: *PSORS1C1*, *BAT2*, *LY6G5C*, *BAT5*, *LY6G6D*, *TNXB*, *CREBL1*, *NOTCH4*, *LOC401252*, *C6orf10*, *BTNL2*, *HLA-DRA*, *HLA-DRB9*, *HLA-DQA1*, and *TAP2*. Most of the hub haplotypes were located in 6p21.33-6p21.32 (MHC III regions) except one from the chromosome 7 (rs11762043).

## Discussion

RA is an inflammatory disease, primarily of the joints, with autoimmune features and a complex genetic component. Among the 411 SNPs we detected from the single-SNP analysis, only five were previously reported to be associated with RA. The other 406 SNPs are candidate SNPs associated with RA. We list the top ten SNPs in the Table 3; the remaining SNPs are summarized at our website [19]. The highlighted SNPs were selected by both the additive and codominant model. Our GWAHI analysis discovered interactions between haplotype blocks, especially 39 hub haplotypes included genes expected to be related to immune response and various signaling pathways. Further study of these genes may give us more insight into the mechanism of RA development.

**Table 3:** Significant SNPs from the SNP association tests

dbSNP ID	Model	*p*-Value	Chr	Chr position	Allele	Strand	Gene symbol	Functional class
**rs2395175^a^**	codominant	2.46 × 10^-112^	6	32,513,004	A/G	+		
**rs660895**	codominant	5.05 × 10^-107^	6	32,685,358	A/G	+		
**rs6910071**	codominant	6.65 × 10^-94^	6	32,390,832	A/G	+	*C6orf10*	intron
**rs2395163**	codominant	1.10 × 10^-90^	6	32,495,787	C/T	+		
**rs3763312**	codominant	1.87 × 10^-86^	6	32,484,326	A/G	+	*BTNL2*	locus
**rs3763309**	codominant	2.06 × 10^-86^	6	32,483,951	A/C	+	*BTNL2*	locus
rs9275224	codominant	5.96 × 10^-86^	6	32,767,856	A/G	+		
rs6457617	codominant	6.83 × 10^-75^	6	32,771,829	C/T	+		
rs9271568	codominant	1.44 × 10^-66^	6	32,698,441	A/G	+		
rs2395185	codominant	8.68 × 10^-65^	6	32,541,145	G/T	+		
**rs660895**	additive	1.40 × 10^-62^	6	32,685,358	A/G	+		
rs9271568	additive	5.33 × 10^-56^	6	32,698,441	A/G	+		
rs2395185	additive	3.30 × 10^-55^	6	32,541,145	G/T	+		
**rs6910071**	additive	7.28 × 10^-55^	6	32,390,832	A/G	+	*C6orf10*	intron
**rs2395163**	additive	2.58 × 10^-54^	6	32,495,787	C/T	+		
rs2516049	additive	4.74 × 10^-53^	6	32,678,378	A/G	-		
rs477515	additive	1.25 × 10^-52^	6	32,677,669	C/T	-		
**rs3763309**	additive	4.34 × 10^-52^	6	32,483,951	A/C	+	*BTNL2*	locus
**rs3763312**	additive	5.29 × 10^-51^	6	32,484,326	A/G	+	*BTNL2*	locus
**rs2395175**	additive	6.53 × 10^-50^	6	32,513,004	A/G	+		

^a^Bold text indicates that the SNPs are selected by both the additive and codominant model.

We provide a new strategy for exploring the interactions among haplotypes in genome-wide association analysis. Our proposed GWAHI narrowed down the set of important haplotype blocks for assessing evidence of interactions using a two-step analysis. For our haplotype interaction analysis, we included not only significant haplotype blocks but also haplotype blocks containing significantly associated SNPs. This could give us more insight of the relationships between the significant SNP and the significant halotypes.

We used the two-step approach to minimize the computational burden by reducing the number of haplotypes. In our analysis, the computational time was reasonable (12 hours using 10 nodes: Dual Core AMD Opteron(TM) Processor 270 CPU, 6 G memory). However, in this two-step approach the haplotype interactions without main effects cannot be detected at Step 1 and thus cannot be included in interaction analysis at Step 2. If we included these haplotypes in interaction analysis, then the computational time would increase exponentially due to the increase of the number of haplotypes. Thus, it is a challenge to handle this computational burden more efficiently for successful GAWHI.

Our haplotype interaction analysis was conducted using the program FAMHAP. The range of permutation *p*-values was dependent on the number of simulations. The *p*-value becomes zero when it is below 1.0 × 10^-4 ^in the default setting (simulation time, 1 × 10^4^). Thus, the rank of the lowest *p*-values (*p*-value = 0) is unattainable. Even though we increased the simulation time to the 1.0 × 10^8^, we still had the same problem. As the simulation time increased, the computing time increased exponentially (from 8 second for the default setting to 8 hours for the 1.01.0 × 10^8 ^simulation time for one run). In order to get higher resolution of the corrected *p*-values, we computed the *p*-values of the chi-square statistics from FAMHAP program by using the chi-square distribution and then applied Bonferroni correction. In fact, the non-zero permutation *p*-values from FAMHAP did not differ much from the corrected *p*-values computed by chi-square statistics.

## List of abbreviations used

GAW: Genetic Analysis Workshop; GWAHI: Genome-wide analysis of haplotype interaction; GWAS: Genome-wide association studies; HLA: Human leukocyte antigen; NARAC: North American Rheumatoid Arthritis Consortium; RA: Rheumatoid arthritis; SNP: Single-nucleotide polymorphism.

## Competing interests

The authors declare that they have no competing interests.

## Authors' contributions

JP carried out the data preprocessing and statistical analysis and drafted the manuscript. JN participated in the study design and helped to draft the manuscript. MJ participated in the data preprocessing. TP conceived of the study, participated in its design and coordination, and helped to draft the manuscript. All authors read and approved the final manuscript.

## Supplementary Material

Additional file 1 Genes matched to KEGG pathways among those identified by haplotype interaction analysis. Click here for file
