# Supplementary material for: Genome-wide analysis of haplotype interaction for the data from the North American Rheumatoid Arthritis Consortium
Source: BMC Proc. 2009 Dec 15;3(Suppl 7):S34. doi: 10.1186/1753-6561-3-s7-s34 (PMC2795932; doi:10.1186/1753-6561-3-s7-s34)
Supplement: Additional file 1 — Genes matched to KEGG pathways among those identified by haplotype interaction analysis. [file 1753-6561-3-S7-S34-S1.pdf]

**Supplemental File 1 - Genes matched to KEGG pathways among those identified by haplotype interaction analysis**

| KEGG pathway | No. genes | Matched genes |
|---|---|---|
| ANTIGEN_PROCESSING_AND_PRESENTATION[a] | 17 | *HLA-C, HLA-DMA, HLA-DMB, HLA-DOA, HLA-DOB, HLA-DPA1, HLA-DQA1, HLA-DQA2, HLA-DQB1, HLA-DQB2, HLA-DRA, HLA-E, HLA-F, HLA-G, LTA, TAP2, TAPBP* |
| TYPE_I_DIABETES_MELLITUS | 16 | *HLA-C, HLA-DMA, HLA-DMB, HLA-DOA, HLA-DOB, HLA-DPA1, HLA-DQA1, HLA-DQA2, HLA-DQB1, HLA-DQB2, HLA-DRA, HLA-E, HLA-F, HLA-G, LTA, TNF* |
| CELL_ADHESION_MOLECULES | 14 | *HLA-C, HLA-DMA, HLA-DMB, HLA-DOA, HLA-DOB, HLA-DPA1, HLA-DQA1, HLA-DQA2, HLA-DQB1, HLA-DQB2, HLA-DRA, HLA-E, HLA-F, HLA-G* |
| NATURAL_KILLER_CELL_MEDIATED_CYTOTOXICITY[a] | 6 | *HLA-C, HLA-E, HLA-G, MICA, MICB, TNF* |
| CALCIUM_SIGNAL_PATHWAY[b] | 3 | *ADCY9, CACNA1C, ITPR3* |
| CELL_COMMUNICATION | 3 | *COL11A2, LAMC3, TNXB* |
| CYTOKINE_CYTOKINE_RECEPTOR_INTERACTION[a] | 3 | *IL2RB, LTA, TNF* |
| ECM_RECEPTOR_INTERACTION | 3 | *COL11A2, LAMC3, TNXB* |
| FOCAL_ADHESION | 3 | *COL11A2, LAMC3, TNXB* |
| GNRH_SIGNALING_PATHWAY[b] | 3 | *ADCY9, CACNA1C, ITPR3* |
| MAPK_SIGNALING_PATHWAY[b] | 3 | *CACNA1C, DAXX, TNF* |
| SMALL_CELL_LUNG_CANCER | 3 | *LAMC3, RXRB, TRAF1* |
| ADIPOCYTOKINE_SIGNALING_PATHWAY[b] | 2 | *RXRB, TNF* |
| ALZHEIMERS_DISEASE | 2 | *LRP1, TNF* |
| CELL_CYCLE | 2 | *CDC25C, MAD1L1* |
| CHOLERA_INFECTION[a] | 2 | *ADCY9, ATP6V1G2* |
| COMPLEMENT_AND_COAGULATION_CASCADES[a] | 2 | *C2, C5* |
| GAP_JUNCTION | 2 | *ADCY9, ITPR3* |
| GLYCAN_STRUCTURES_BIOSYNTHESIS_1 | 2 | *ALG12, GALNT2* |
| HEMATOPOIETIC_CELL_LINEAGE | 2 | *HLA-DRA, TNF* |
| LONG_TERM_POTENTIATION | 2 | *CACNA1C, ITPR3* |
| PURINE_METABOLISM | 2 | *ADCY9, ZNRD1* |
| TASTE_TRANSDUCTION | 2 | *ITPR3, KCNB1* |

| | | |
|---|---|---|
| TYPE_II_DIABETES_MELLITUS | 2 | *CACNA1C, TNF* |
| FATTY_ACID_ELONGATION_IN_MITOCHONDRIA | 1 | *PPT2* |
| ANDROGEN_AND_ESTROGEN_METABOLISM | 1 | *HSD17B8* |
| OXIDATIVE_PHOSPHORYLATION | 1 | *ATP6V1G2* |
| PYRIMIDINE_METABOLISM | 1 | *ZNRD1* |
| N_GLYCAN_BIOSYNTHESIS | 1 | *ALG12* |
| O_GLYCAN_BIOSYNTHESIS | 1 | *GALNT2* |
| GLYCEROLIPID_METABOLISM | 1 | *AGPAT1* |
| GLYCEROPHOSPHOLIPID_METABOLISM | 1 | *AGPAT1* |
| ETHER_LIPID_METABOLISM | 1 | *AGPAT1* |
| NITROGEN_METABOLISM | 1 | *CA5A* |
| NEURODEGENERATIVE_DISEASES | 1 | *NEFH* |
| ABC_TRANSPORTERS_GENERAL | 1 | *TAP2* |
| RIBOSOME | 1 | *RPS18* |
| RNA_POLYMERASE | 1 | *ZNRD1* |
| PPAR_SIGNALING_PATHWAY[b] | 1 | *RXRB* |
| PHOSPHATIDYLINOSITOL_SIGNALING_SYSTEM | 1 | *ITPR3* |
| NEUROACTIVE_LIGAND_RECEPTOR_INTERACTION | 1 | *GRIK3* |
| APOPTOSIS | 1 | *TNF* |
| DORSO_VENTRAL_AXIS_FORMATION | 1 | *NOTCH4* |
| NOTCH_SIGNALING_PATHWAY[b] | 1 | *NOTCH4* |
| TGF_BETA_SIGNALING_PATHWAY[b] | 1 | *TNF* |
| AXON_GUIDANCE | 1 | *SRGAP2* |
| TOLL_LIKE_RECEPTOR_SIGNALING_PATHWAY[b] | 1 | *TNF* |
| JAK_STAT_SIGNALING_PATHWAY[b] | 1 | *IL2RB* |
| T_CELL_RECEPTOR_SIGNALING_PATHWAY[b] | 1 | *TNF* |
| FC_EPSILON_RI_SIGNALING_PATHWAY[b] | 1 | *TNF* |
| LONG_TERM_DEPRESSION | 1 | *ITPR3* |
| REGULATION_OF_ACTIN_CYTOSKELETON | 1 | *TIAM1* |
| INSULIN_SIGNALING_PATHWAY[b] | 1 | *FLOT1* |
| MELANOGENESIS_ | 1 | *ADCY9* |
| AMYOTROPHIC_LATERAL_SCLEROSIS | 1 | *NEFH* |
| PRION_DISEASE | 1 | *TNF* |
| EPITHELIAL_CELL_SIGNALING_IN_HELICOBACTER_ PYLORI_INFECTION[b] | 1 | *ATP6V1G2* |
| THYROID_CANCER | 1 | *RXRB* |
| NON_SMALL_CELL_LUNG_CANCER | 1 | *RXRB* |

[a]Immune responses

[b]Signaling-pathways
